# Isolation and Characterization of Three Cassava Elongation Factor 1 Alpha (MeEF1A) Promoters

**DOI:** 10.1371/journal.pone.0084692

**Published:** 2014-01-03

**Authors:** Sony Suhandono, Ardha Apriyanto, Nisa Ihsani

**Affiliations:** School of Life Sciences and Technology, Institut Teknologi Bandung, Bandung, Jawa Barat, Indonesia; University Paris South, France

## Abstract

In plant genetic engineering, the identification of gene promoters leading to particular expression patterns is crucial for the development of new genetically modified plant generations. This research was conducted in order to isolate and characterize several new promoters from cassava (*Manihot esculenta* Crantz) elongation factor 1 alpha (EF1A) gene family. Three promoters MeEF1A3, MeEF1A4 and MeEF1A5 were successfully isolated. Sequence analyses showed that all of the promoters contain three conserved putative cis-acting elements which are located upstream of the transcription start site. These elements are included a TEF1, a TELO and TATA boxes. In addition, all of the promoters also have the 5′UTR intron but with a different lengths. These promoters were constructed translationally with gus*A* reporter gene (promoter::*gusA* fusion) in pBI-121 binary vector to build a new binary vector using Overlap Extension PCR Cloning (OEPC) technique. Transient expression assay that was done by using agroinfiltration method was used to show functionality of these promoters. Qualitative and quantitative analysis from GUS assay showed that these promoters were functional and conferred a specific activity in tobacco seedlings (*Nicotiana tabacum*), tomato fruits (*Solanum lycopersicum*) and banana fruits (*Musa acuminata*). We hypothesized that MeEF1A6 could be categorized as a constitutive promoter because it was able to drive the gene expression in all transformed tissue described in here and also comparable to CaMV35S. On the other hand, MeEF1A3 drove specific expression in the aerial parts of seedlings such as hypocotyl and cotyledon thus MeEF1A5 drove specific expression in fruit tissue. The results obtained from transient analysis showed that these promoters had a distinct activity although they came from same gene family. The DNA sequences identified here are new promoters potentially use for genetic engineering in cassava or other plants.

## Introduction

Cassava (*Manihot esculenta* Crantz) is a very important tropical food crop for approximately 800 million people around the world [1]. As a result, the demand for cassava is also increasing. One of the efforts to increase the cassava production is by developing new cassava varieties which are adapted to a various agroclimate condition and tolerant to climate change. Classical breeding of cassava is not easy and may take years. This is also not feasible due to self-incompatibility, poor flowering ability, low pollen fertility and low fruit set rate [2]. One possible solution is the use of genetic modification to introduce gene of interest with important agronomic traits such as disease resistance, abiotic stress tolerant, extended shelf life (post-harvest-deterioration), low cyanogen content and increase nutritional value (e.g., vitamin A, Zn, Fe) content [1], [3]. This is promising because protocols for stable genetic modification of cassava have been successfully established by several research groups [4], [5]. However, the identification of gene promoters leading to particular expression patterns is also crucial for the development of new genetically modified plant generations.

One of interesting protein is eukaryotic elongation factor 1 alpha (eEF1A), which is an important component for protein biosynthesis [6]. eEF1A catalyzes the binding of aminoacyl-tRNA to the A-site of the ribosome by a GTP-dependent mechanism [7]. eEF1A constitutes up to 3–10% of the total soluble protein and is considered as one of the most abundant soluble protein in cells cytoplasm [8]. Besides its canonical role in protein biosynthesis, several other activities have been described for this protein (so called moonlighting protein) [9], namely interaction with valyl-tRNA synthetase complex [10], actin [11], tubulin [12], ubiquitin [13] and calmodulin [14]. Moreover, eEF1A was reported to be involved in signal transduction [15], [16], virus infection mechanism [17], nuclear export of proteins [18], and mitochondrial tRNA import [19]. It is also suspected to have a role in apoptosis [9], DNA replication/repair protein networks regulation [20], heat shock proteins regulation [21] and has a molecular chaperone-like activity [22], [23].

Many studies revealed that eEF1As are typically encoded by multigene family [24]–[29], a fact shared in cassava [30]. In plants, one gene family may comprise of two to twenty copies of eEFIA. For example, soybean [31] and carrot [32] contain two copies; *Oryza sativa* [26] and *Arabidopsis thaliana* [24] have four copies each, and sugarcane may contain up to twenty copies [29].

The genes encoding eEF1A are highly expressed in all developing tissues, which exhibit high levels of protein synthesis. However, several studies revealed that expression of the eEF1A genes may be varies during developmental stages [29], [32]–[34], low temperature [35], high temperature [36], [37], drought [38], light [31], low oxygen [39], chemical induction (e.g ethepon) [40], pathogen attack [41] and physical wounding [42]. Our previous study showed that one of the eEF1A genes in cassava (MeEF1A1) was expressed in early stages of plant development and also induced by wounding [43]. Recent study in *A.thaliana* using microarray technique showed that eEF1A gene family was expressed in all tissues but it was also indicated that each eEF1A genes had a unique expression pattern regulated differently by a variety of stimuli [44].

Although eEF1A genes in some plant species have been well characterized, the cassava eEF1A genes family member, especially their expression and promoter activity have not been reported before and thus need to be explored. In this study, we describe the isolation and functional characterization of several new promoters of *EF1A* gene family from *Manihot esculenta* (MeEF1A). The MeEF1A promoters were analyzed by transient expression system using GUS reporter gene in both dicot and monocot plants such as tobacco (*Nicotiana tabacum),* tomato (*Solanum lycopersicum*) and banana (*Musa acuminata).* We hope these promoters may have a unique characteristic and can be used for genetic engineering in plant.

## Materials and Methods

### Materials

Cassava (*Manihot esculenta* Cranz var. Adira) leaf materials were used for promoter isolation. Plants such as Tobacco (*Nicotiana tabacum*) seedling, Tomato (*Solanum lycopersicum*) and Banana (*Musa acuminata* var. Mas) fruit materials were used for expression analysis.

### Promoter Isolation

Gene family identification was done by comparing the first exon of EF1A gene from cassava (AF041463) using blastn to the EF1A gene family available on *Manihot esculenta* genome database (Phytozome) [45]. A set of primers then was designed from that blastn result in order to clone the promoters from EF1A gene family. Genomic DNA was isolated from cassava leaves using CTAB method [46]. The promoter regions from each gene family were amplified using specific primers (Table 1). PCR amplification was performed using Kapa 2G Polymerase™ (Kapa Biosystem) in Veriti 96 well Thermal cycler (Applied Biosystems).

**Table 1:** Primer sequences used in this study.

No	Primer Name	Sequences (5′-3′)	Base
		**Cloning**	
**1**	MeEF1A-UnivReverse	GTGAACCTTCTCITTACCCATT	22
**2**	MeEF1A3-Forward	TTTACCGTTGTTGGCAGCAA	20
**3**	MeEF1A5-Forward	AATTCTTTCCCTGCGCCAAT	20
**4**	MeEF1A6-Forward	AAAGATGGACGGCAAATGGT	20
		**Overlap Extension PCR Cloning**	
**5**	OEPC-UnivReverse	*AGGACGTAACATAAGGGACTGACCACCCGG* **GTGAACCTTCTCGTTACCCTTT**	52
**6**	OEPC-MeEF1A3Forward	*CATGATTACGCCAAGCTTGCATGCCTGCAG* **TTTACCGTTGTTGGCAGCA**	49
**7**	OEPC-MeEF1A5Forward	*CATGATTACGCCAAGCTTGCATGCCTGCAG* **AATTCTTTCCCTGCGCCAAT**	50
**8**	OEPC-MeEF1A6Forward	*CATGATTACGCCAAGCTTGCATGCCTGCAG* **AAAGATGGACGGCAAATGGT**	50
		**Validation**	
**9**	CaMV 35-Forward	ATAGAGGACCTAACAGAACTCGC	23
**10**	GUS Seq-Reverse	GGCTTTCTTGTAACGCGC	18

Bold nucleotides are the specific primers to the promoter therefore italic nucleotides are the specific primer complimentary to the vector.

Each of PCR products from single PCR reaction were purified using Geneaid™ PCR purification kit (Geneaid) following the manufacturer’s protocol and cloned into pJET1.2/blunt vector (Fermentas). Then the vectors are introduced into *Escherichia coli* strain DH5α with heat shock method [47]. The plasmid vector was extracted by Geneaid™ plasmid isolation kit (Geneaid) and both strands were sequenced using pJET 1.2 forward and pJET 1.2 reverse primers at Macrogen Inc, South Korea.

### Promoter Sequence Analysis

The sequence data set were analysed using Geneious™ 5 software [48] while the homology searches were performed using blastn at the NCBI website (http://blast.ncbi.nlm.nih.gov). Subsequently, a blastn search in cassava genome was performed at the Phytozome v9.0 website (http://www.phytozome.net/cgi-bin/gbrowse/cassava) [49]. Promoter region was confirmed using Expressed Sequence Tag (EST) database from GenBank [50] and prediction of 5′UTR (Untranslated Region) intron was performed at NetGene2 website (http://www.cbs.dtu.dk/services/NetGene2/) [51]. Conserved cis-acting regulatory was carried out using the PATTERN search from Softberry website (http://www.softberry.com/berry.phtml). PLACE [52] and PlantCARE [53] software were used to detect putative cis-acting regulatory elements in the MeEF1A promoter sequence, The promoter architecture was drawn using CLC Sequence Viewer™ 6 software (http://www.clcbio.com).

### Construction of Plant Transformation Vectors

**Figure 1:**
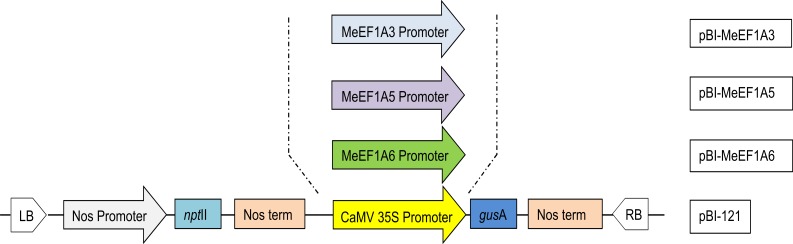
Schematic diagram of three MeEF1A promoters constructs. The region showed in here represent T-DNA region in pBI-121.

The binary vector pBI-121 [54] was used in this study. The isolated promoters were inserted into this binary vector and replacing the CaMV 35S promoter. Diagrammatical construct can be seen in Figure 1. The construction of plant transformation vector was generated by using Overlap extension PCR cloning (OEPC) method [55]. In present study we used Phusion Polymerase (Finnzymes). Specific OEPC primer for constructing the vectors can be seen in Table 1. In order to verify the correct integration, the plasmids were sequenced using each specific promoter forward primer and gus-Seq R primer (Table 1).

### Transient Transformation of Plant Tissue

The expression vector constructs pBI-MeEF1A3, pBI-MeEF1A5, pBI-MeEF1A6 and pBI- 121 were introduced into *Agrobacterium tumefaciens* strain GV3101 by freeze-thaw method [56]. To investigate the promoter’s activity, one week tobacco seedlings, mature tomato and banana fruit tissues were transformed using these agrobacteria lines (namely transient transformation lines). Agrobacteria lines were grown as individual culture at room temperature (27°C) in YEP medium containing antibiotic selection (100 mg/ml Kanamycin and 50 mg/ml Rifampicin) until each culture reached OD_600_ = 0.8. Individual cultures were centrifuged at 7,000 g for 10 min and suspended in infiltration media [0.5× MS (pH 6.0, Caisson Laboratories), 1% sucrose, 100 µM acetosyringone, 0.005% Silwet L-77]. The plant tissues were submerged in a clean petri dish containing 20 ml of each suspension culture under vacuum (Biorad Vacuum Pump) for 15 minutes. Co-cultivation was carried out in the dark at 23°C for 72 hr. Quantitative and qualitative measurements of GUS activity were performed post co-cultivation. The pBI-121 binary vector that contains the GUS gene driven by the CaMV 35S promoter was used as a positive control and *A.tumefaciens* without expression vector was used as a negative control.

### Qualitative Analysis of GUS Activity

Expression of the *β*-glucuronidase (GUS) gene was detected by histochemical staining [57]. All of transformed tissue samples (including positive and negative control) were immersed in X-Gluc solution (1 mM 5-bromo-4-chloro-3-indolyl-*β*-D-glucuronide; Sigma), 100 mM sodium phosphate buffer pH 7.0, 0.5 mM K_3_[Fe(CN)_6_], 0.5 mM K_4_[Fe(CN)_6_], 10 mM Na_2_EDTA, 0.1% (v/v), Triton X-100 and incubated at 37°C for 18 h in the dark. For better visualization of the stained tissue, the samples were rinsed at room temperature three times with increasingly concentrated ethanol solutions (70–100%) in order to remove chlorophyll. The cleared samples were observed and photographed with a SLR digital camera (Canon EOS 1100DC). GUS-stained tissues and plants in the present paper represent the typical results of at least three independent transient transformation lines for each construct. Each of independent experiment lines was consist of 30 samples.

### Quantitative Analysis of GUS Activity

Banana and tomato tissue from transformed and untransformed samples were used to determine GUS activity. Approximately of 200 mg of tissue were ground in a mortar with liquid nitrogen and homogenized in 200 µL of GUS extraction buffer (50 mM phosphate buffer, pH 7.0, 10 mM Na_2_EDTA, 0.1% Triton X-100, 0.1% sodium lauryl sarcosine and 10 mM *β*-mercaptoethanol. The homogenate was then centrifuged for 10 min at 12.000 g at 4°C, and the quantification of GUS activity in the supernatants was determined according to a previously described method [57], [58]. Briefly, GUS activity assay was performed using PNPG (p-nitrophenyl-D-glucuronide) as substrate and was measured at 415 nm with a Ultrospec 2000/UV apparatus (Pharmacia Biotech) and expressed as nmol of PNP (p-nitrophenyl) released per min per mg of protein at 37°C. Protein concentration of the samples was determined by the Bradford assay method using BSA (bovine serum albumin) as standard [59]. Bradford assay was repeated three times. The data presented were collected from at least three independent transient transformation lines for each construct. Each of independent experiment lines was consist of 10 samples. Differences in GUS activity among treatment groups were tested with least significant difference (LSD) and one-way analysis of variance (ANOVA) in GenStat 15.0 software.

## Results

### Isolation and Sequence Analysis of MeEF1A Promoters

The three fragments from genomic DNA of *M.esculenta* cultivar Adira were succesfully cloned and sequenced, a 1247 bp named MeEF1A3, 1254 bp named MeEF1A5 and a 1168 bp named MeEF1A6. In order to identify these fragments, the sequences were analyzed using the blastn on Cassava Phytozome v9.0 website [49]. A homology search at that website resulted in a highly homologous sequence present in scaffold 02421 for MeEF1A5 (99%) and for MeEF1A6 (99%), but in scaffold 03015 we found only 90% similarity for MeEF1A3.

These sequences were also compared with the EST’s of *M. esculenta* in order to identify putative 5′UTR intron which may present in these promoters. The promoters MeEF1A3 and MeEF1A6 had 99% similarity with an EST from cassava (DB934938) and (DB936919), respectively. A 496 bp intron was found in the 5′UTR of MeEF1A3 and a 842 bp intron was found in the the 5′UTR of MeEF1A6. Only MeEF1A5 that didn’t have a very high EST similarity in GenBank database (about 98% with DB9812938). Moreover, we only found one splicing site from the EST database, therefore the 5′UTR intron was predicted using splicing site NetGene2 software [51] and we found a putative 856 bp intron in the 5′UTR of MeEF1A5.

Sequence analyses showed that EF1A promoters have putative cis-acting elements which are predicted based on the sequence similarity and the relative position to the transcription start site (TSS) as we can see on (Table 2). These putative control elements that contain a TEF1 box, a TELO box and TATA box are conserved among eEF1A promoter in plants (Table 2). These putative element sequences in MeEF13, MeEF1A5 and MeEF1A6 are similar to the consensus sequences (Table 2).

**Table 2:** Conserved motif elements in plants EF1A promoters.

No	Promoter	TEF1 Box		TELO Box		TATA Box		TATA Box		TSS	Reference
			n		n	1st	n	2nd	n		
1	SoEF1A1	TTGGGCCCAATAGCCC	13	TGAACCCTAG	9	TTATAAAAA			36	TCGGCCG	AF331849,JN132399 [64]
2	SlEF1A1	AGGGGCATTTACGTAA	27	TGAACCCTAA	15	CTATAAAAT			26	TTCATTA	X53043 [63]
3	AtEF1A1	AGGGGCATAATGGTAA	25	TAAACCCTAA	21	CTATAAATA			20	TCCATTT	X16430 [62]
4	AtEF1A2	AAGGGTAAAATTGTCA	20	TAAACCCTAA	18	CTATAAATA			21	TTTATTT	X16431 [62]
5	AtEF1A3	AGGGGTACGTTTGTAA	20	TAAACCCTAA	24	CTATAAATA			5	CTCGAAT	X16432 [62]
6	AtEF1A4	AAGGGCAAATTAGTAA	24	AAAACCCTAG	11	CTATAAGTA			18	TTAGGGT	X16432 [62]
7	MeEF1A1	AGGGTCAAAAATGTAA	39	GTAACCCTAA	9	CTATATATA	1	GTATAAGTA	16	CTCAGTT	AF041463 [30]
8	MeEF1A3	AAGGGCAAAACCGTAA	30	GTAACCCTAG	11	CTATAAATA			26	GTTCGCA	KC955123 [This study]
9	MeEF1A5	TTGGACAAAATCGTAA	23	GGAACCCTAA	11	CTATAAATA			28	TTGTTTC	KC955124 [This study]
10	MeEF1A6	TTAGACAAAACCGTAA	11	GGAACCCTAG	11	CTATAAATA			28	TCCCGCT	KC955125 [This study]
	Consensus	WNGGWCAAAANNGTAA		GNAACCCTAR		CTATAAATA					

Bold nucleotides are conserved nucleotides.

n: nucleotides between motifs.

**Figure 2:**
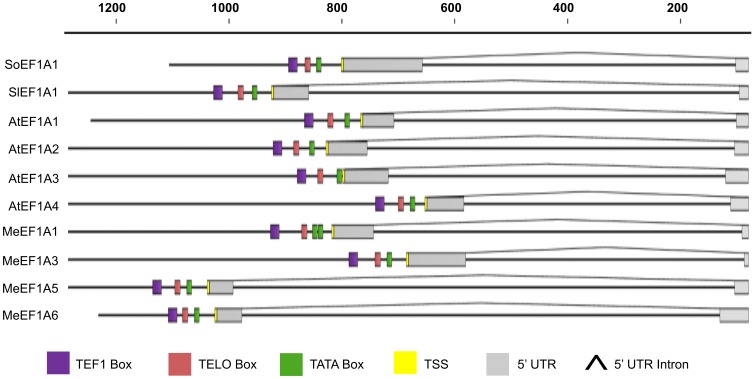
The elongation factor 1 alpha promoter architecture in plants. Nucleotides number relative to their start codon (ATG) show on top of the graph. SoEF1A1: *Saccharum officinarum* EF1A1 promoter (AF331849, JN132399); SlEF1A1: *Solanum lycopersicum* EF1A1 (X53043); AtEF1A1: *Arabidopsis thaliana* EF1A1 promoter (X16430); AtEF1A2: *Arabidopsis thaliana* EF1A2 promoter (X16431); AtEF1A3: *Arabidopsis thaliana* EF1A3 promoter (X16432); AtEF1A4: *Arabidopsis thaliana* EF1A4 promoter (X16432); MeEF1A1: Manihot esculenta EF1A1 promoter (AF041463); MeEF1A3: Manihot esculenta EF1A3 promoter (KC955123); MeEF1A5: Manihot esculenta EF1A1 promoter (KC955124); MeEF1A6: Manihot esculenta EF1A1 promoter (KC955125).

The summary of several plants EF1A promoter architecture including our results can be seen on Figure 2. The nucleotide sequence and annotation of MeEF1A3, MeEF1A5, and MeEF1A6 promoters were deposited into GenBank [50] under accession number KC9551253, KC9551254 and KC955125, respectively.

### Qualitative Analysis of GUS Activity

**Figure 3:**
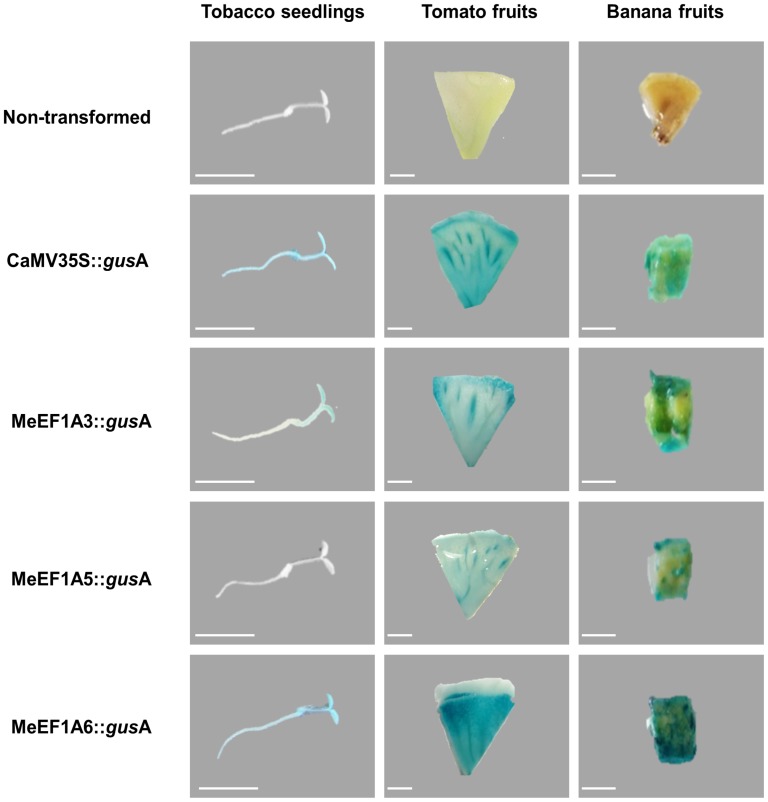
Photographic representations of the comparison of transient histochemical assay in various tissue carrying gusA gene driven by different MeEF1A promoters. Scale bars indicated 5

The activities of MeEF1A promoters were evaluated through transient expression study by using agro infiltration method in various plant tissues. The results from GUS histochemical assay revealed that MeEF1A3 was able to drive the expression of gusA gene in seedlings cotyledon but not in its root, MeEF1A6 able to regulate the gusA gene in almost all of tobacco seedlings tissue, but interestingly MeEF1A5 was not able to regulate the gusA gene in all of seedling tissue (Figure 3). In addition, MeEF1A3, MeEF1A5 and MeEF1A6 were able to drive the expression of gus*A* gene in banana fruit pulp and tomato fruit with different expression level (Figure 3). As expected, the CaMV35S promoter drives the expression of gusA gene in all of tissues (tobacco seedlings, banana fruit and tomato fruit). Furthermore, no endogenous GUS activity was detected in non-transformed tissues (negative control). This observation clearly indicated that the blue spots observed were due to introduced genes.

### Quantitative Analysis of GUS Activity

**Figure 4:**
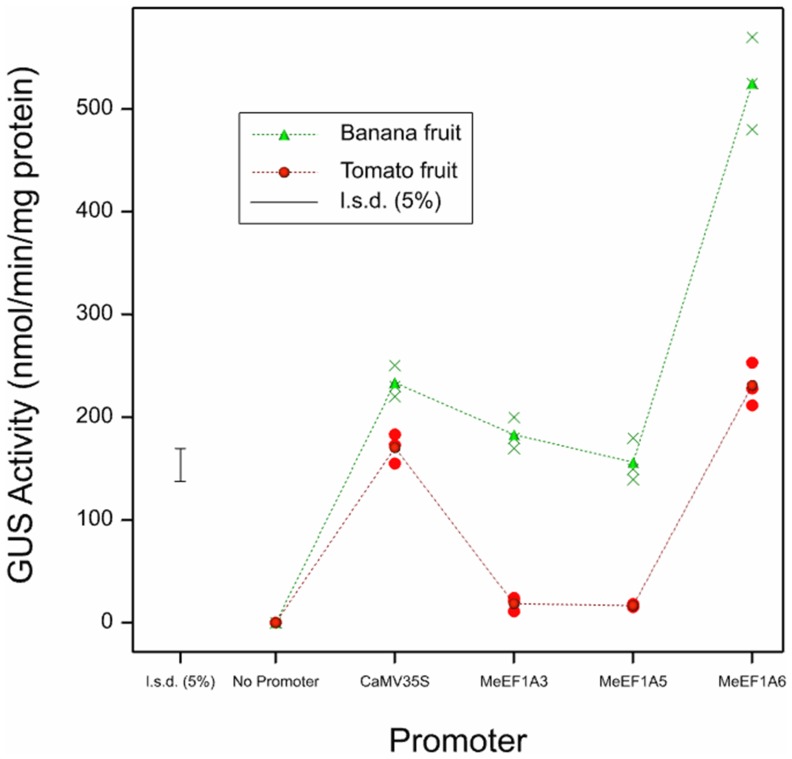
GUS activities in monocot and dicot fruit system driven by different MeEF1A promoters. The quantification of GUS activity for each promoter construct was replicated three times. Statistical analysis was performed using least significant difference and homogeneity of variance test by GenStat 15.0, and one way ANOVA test was used for the statically analysis.

To determine the strengths of the different MeEF1A promoters, a quantitative GUS activity assay was conducted by spectrophotometric PNPG assay. Measurement of GUS enzyme activity in this study was made on monocot (banana) and dicot (tomato) system (not in tobacco seedlings). The spectrophotometric data for the transformed tomato and banana fruits containing different MeEF1A constructs are shown in Figure 4.

Varying levels of GUS activities were obtained among fruits transformed with the different promoter constructs (Figure 4). There were significant differences among tomato and banana fruits. Overall, the GUS activity gave higher value in banana fruits. In this fruit, MeEF1A6 exhibited the highest expression level with an average value of 525 nmol/min/mg whereas GUS activity driven by CaMV35S promoter using similar tissue was 234 nmol/min/mg (two-fold lower). Other promoters MeEF1A3 and MeEF1A5 had no significant differences and had relatively equal strength compared to CaMV35S promoter. This result showed that MeEF1A6 promoter was stronger than CaMV35S promoter in banana fruit.

In tomato fruit, MeEF1A6 promoter drove a higher expression than CaMV35S promoter. However, the level of expression was not as high as in banana fruits. In the other hands, MeEF1A3 and MeEF1A5 had very low activity compared with MeEF1A6 and CaMV35S in tomato fruit but their activity were no significant differences in this tissue (Figure 4). All of these quantitative data correlated with the results observed in the histochemical GUS staining patterns in both banana and tomato fruit tissues (Figure 3).

## Discussion

The MeEF1A5 and MeEF1A6 promoter sequence showed high similarity with the scaffolds data (02421) obtained from Cassava Phytozome database, 99% and 99% identity, respectively. These differences are probably because we were using Adira genotypes from BALITKABI, Indonesia instead of AM560-2 genotypes developed by CIAT, Columbia [45]. The same results were reported by other cassava researcher when the sequences were isolated from different genetic background [60]. Interestingly, MeEF1A3 promoter had 90% similarity to corresponding sequence at the scaffold 03015 from database. This was because we found 108 bp deletions in MeEF1A3 promoter region. Some research reported that insertion or deletion that occurred in promoter region was not only connected with improper gene regulation that leads to disease but also as a part of micro-evolution process as reviewed in Vedel and Scotti [61].

It is interesting that introns were found on the 5′-end of the MeEF1A3, MeEF1A5 and MeEF1A6 promoters. The promoter sequences of the EF1A gene family (AtEF1A1-4) from Arabidopsis [62], SlEF1A1 [63] from tomato and SoEF1A1 [64] from sugarcane also have 5′UTR intron. This data indicated that the present of 5′ UTR intron were conserved among EF1A promoters in plant (Figure 2). The presence of intron in 5′UTR region has been shown to increase the levels of gene expression EF1A in *A.thaliana* [62]. This phenomenon is referred as IME or Intron-Mediated Enhancement [65]. However, the mechanism is largely unknown. Some efficiently spliced introns boost expression more than 10-fold, while others have little or no effect [66]. Our previous results using the promoter MeEF1A1 also showed that the presence of 5′UTR intron affects the gene expression level [30].

Overall, the highest promoter activity was conferred by MeEF1A6 and the lowest was conferred by MeEF1A5 (Figure 4). Compared to the other MeEF1A promoters, MeEF1A5 had the biggest putative 5′UTR intron size (856 bp) and MeEF1A3 had the lowest putative 5′UTR intron size (490 bp) (Figure 2). Eventhough recent study had revealed that the 5′UTR intron had a role in enhancing the expression of the genes, however the greater size of the 5′UTR intron was not correlated to higher gene expression, and vice versa [67].

The results from 5′UTR splicing site analyses showed that all of the promoters had conserved splicing site donor AG/GTA and splicing acceptor site CAG/AT. It is also corroborate with what has been previously reported that the conserved splicing donor site in plants is AG/GTAAG while its acceptor TGCAG/G [68]. This result indicated that 5′UTR splicing mechanism among plants EF1A gene family were conserved.

Another interesting feature in MeEF1A promoters is that they had several conserved putative cis-acting element such as TATA, TELO and TEF1 boxes at similar arrangement as we can see from Table 2. Cis-acting regulatory elements are important molecular switches involved in the transcriptional regulation of dynamic networks of gene activities controlling various biological processes. A putative TATA box (consensus CTATAWATA) sequence was located at a region approximately between –10 and –45 relative to the transcription start site (TSS). All of plants EF1A promoters have single putative TATA box except for MeEF1A1 that contain two putative TATA boxes. Another motif that we found is TELO box (consensus AACCCTA). This element was known to interact with AtPurα in *A. thaliana* [69]. AtPurα is Purα homolog protein, a conserved multifunctional protein in eukaryote and play an important role for activating or repressing transcription and translation [70]. TELO box usually found in the upstream regulatory of ribosomal protein and other translational related gene [71]. It drives gene expression in root primordial [72]. The TELO box alone does not confer specificity and must act with other elements such as TEF1 box to drive expression in root meristem [72]. This sequence was located approximately between −20 and −55 relative to TSS.

The last cis-acting motif that we found is TEF1 box (consensus ARGGRYANNNNNGTAA). This element was initially identified in *A. thaliana* EF1A gene [62] and several Arabidopsis RP genes [73]. This motif was located between −40 and −110 relative to TSS. TEF1 box is the target for two heteromeric protein complexes C1 and C2 [74]. Unlike the TELO box, the TEF1 box alone can confer specific expression, activating transcription in cells entering cell cycle, undergoing the transition from quiescent to mitotically active stages [73]. However, the TELO box is usually associated and works synergically with TEF1 box or Motif Site II which active in mersitematical tissue or dividing cell [69]. From the results we can see that MeEF1A6 was active in all of the tissue tested in here therefore MeEF1A3 and MeEF1A5 can only drove the expression in specific tissue (Figure 3; 4) although they had similar conserved putative cis-acting element (as described below).

Generally, the transgene-promoter activities in the plants were affected by the compatibility between the promoter and the type of plant and the activity of transcription factors to bind to a specific subset of a promoter [75]. All of these promoters had TATA box, TELO Box and TEF1 Box at different positions and also had different 5′UTR intron length (Figure 2). This was likely to be the most important factor that made a different expression pattern in these promoters. We believe that the cis-acting motif other than conserved cis-acting motif which described in here may also take a role. Further investigation needs to be done using promoter deletion analysis for characterizing of these promoters such as previously conducted by Curie et al [76] in *A.thaliana* EF1A promoter (AtEF1A1).

## Conclusions

In this study, the isolation and characterization of three promoter sequences of MeEF1A gene coding for the elongation factor 1 alpha protein of cassava were reported. Results obtained from transient expression experiments in tobacco seedlings (*Nicotiana tabacum*), tomato fruits (*Solanum lycopersicum*) and banana fruits (*Musa acuminata*) showed that these promoter sequences are functional, and therefore, it is suitable for further experiments, including stable genetic transformation of model plants (e.g *A.thaliana*) to characterize these promoters. In addition, the results obtained from sequence comparative analysis showed that promoter activity from a gene family had a distinct activity although they have similar arrangement of conserved cis-acting motifs. Based solely on our result, we hypothesized that MeEF1A6 may fell under the category of constitutive promoter and also comparable to CaMV 35S promoter therefore MeEF1A3 and MeEF1A5 were specific promoter (non-constitutive promoter). In summary, the DNA sequence identified here is a new promoter that can be a potential candidate for genetic engineering of cassava or other plant.
