# Correction: Isolation and Characterization of Three Cassava Elongation Factor 1 Alpha (MeEF1A) Promoters

**DOI:** 10.1371/journal.pone.0117871

**Published:** 2015-02-03

**Authors:** The PLOS ONE Staff

There is an error in the third sentence of the Abstract. The correct sentence is: Three promoters MeEF1A3, MeEF1A5 and MeEF1A6 were successfully isolated.

There is an error in the legend for Fig. 2. Please see the complete, corrected Fig. 2 here.

**Figure 2:**
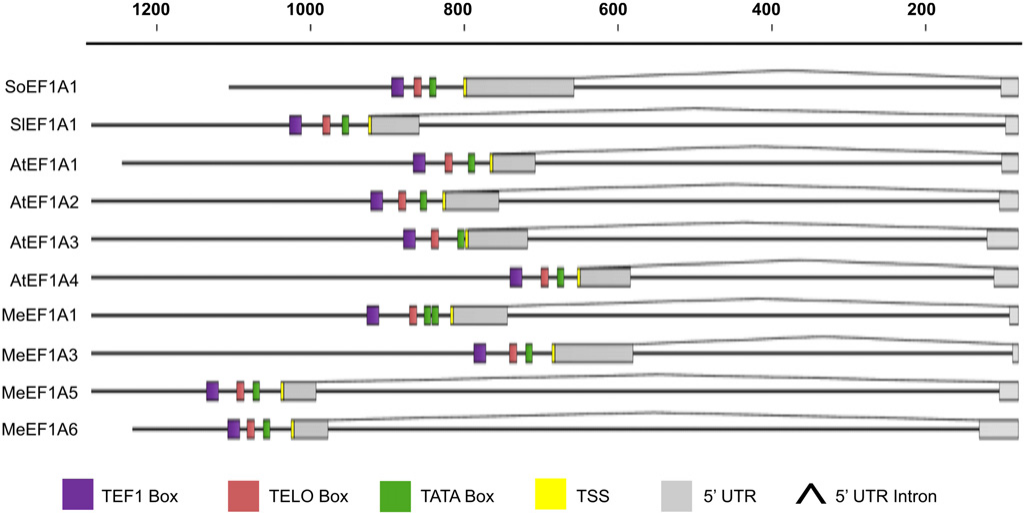
The elongation factor 1 alpha promoter architecture in plants. Nucleotides number relative to their start codon (ATG) show on top of the graph. SoEF1A1: *Saccharum officinarum* EF1A1 promoter (AF331849, JN132399); SlEF1A1: *Solanum lycopersicum* EF1A1 (X53043); AtEF1A1: *Arabidopsis thaliana* EF1A1 promoter (X16430); AtEF1A2: *Arabidopsis thaliana* EF1A2 promoter (X16431); AtEF1A3: *Arabidopsis thaliana* EF1A3 promoter (X16432); AtEF1A4: *Arabidopsis thaliana* EF1A4 promoter (X16432); MeEF1A1: Manihot esculenta EF1A1 promoter (AF041463); MeEF1A3: Manihot esculenta EF1A3 promoter (KC955123); MeEF1A5: Manihot esculenta EF1A5 promoter (KC955124); MeEF1A6: Manihot esculenta EF1A6 promoter (KC955125).
